# Age-Related Anabolic Resistance of Myofibrillar Protein Synthesis Is Exacerbated in Obese Inactive Individuals

**DOI:** 10.1210/jc.2017-00869

**Published:** 2017-07-14

**Authors:** Benoit Smeuninx, James Mckendry, Daisy Wilson, Una Martin, Leigh Breen

**Affiliations:** 1 School of Sport, Exercise and Rehabilitation Sciences, University of Birmingham, Edgbaston, Birmingham B15 2TT, United Kingdom; 2 MRC Arthritis Research UK Centre for Musculoskeletal Ageing Research, University of Birmingham, Edgbaston, Birmingham B15 2TT, United Kingdom; 3 Queen Elizabeth Hospital Birmingham, University Hospitals Birmingham NHS Foundation Trust, Edgbaston, Birmingham B15 2TT, United Kingdom; 4 Institute of Clinical Sciences, University of Birmingham, Edgbaston, Birmingham B15 2TT, United Kingdom

## Abstract

**Context:**

A diminished muscle anabolic response to protein nutrition may underpin age-associated muscle loss.

**Objective:**

To determine how chronological and biological aging influence myofibrillar protein synthesis (MyoPS).

**Design:**

Cross-sectional comparison.

**Setting:**

Clinical research facility.

**Participants:**

Ten older lean [OL: 71.7 ± 6 years; body mass index (BMI) ≤25 kg ⋅ m^−2^], 7 older obese (OO: 69.1 ± 2 years; BMI ≥30 kg ⋅ m^−2^), and 18 young lean (YL) individuals (25.5 ± 4 years; BMI ≤25 kg ⋅ m^−2^).

**Intervention:**

Skeletal muscle biopsies obtained during a primed-continuous infusion of l-[ring-^13^C_6_]-phenylalanine.

**Main Outcome Measures:**

Anthropometrics, insulin resistance, inflammatory markers, habitual diet, physical activity, MyoPS rates, and fiber-type characteristics.

**Results:**

Fat mass, insulin resistance, inflammation, and type II fiber intramyocellular lipid were greater, and daily step count lower, in OO compared with YL and OL. Postprandial MyoPS rates rose above postabsorptive values by ∼81% in YL (*P* < 0.001), ∼38% in OL (*P* = 0.002, not different from YL), and ∼9% in OO (*P* = 0.11). Delta change in postprandial MyoPS from postabsorptive values was greater in YL compared with OL (*P* = 0.032) and OO (*P* < 0.001). Absolute postprandial MyoPS rates and delta postprandial MyoPS change were associated with step count (*r*^2^ = 0.33; *P* = 0.015) and leg fat mass (*r*^2^ = 0.4; *P* = 0.006), respectively, in older individuals. Paradoxically, lean mass was similar between groups, and muscle fiber area was greater in OO vs OL (*P* = 0.002).

**Conclusion:**

Age-related muscle anabolic resistance is exacerbated in obese inactive individuals, with no apparent detriment to muscle mass.

## Teaser

We studied how chronological and biological aging influence muscle anabolism and found that nutrient-stimulated muscle protein synthesis rates are impaired in obese, inactive older individuals.

Age-related muscle loss [sarcopenia (1)] impairs physical function, increases the risk of falls/fractures and metabolic disease, and is associated with mortality (2–5). As the number of older individuals globally rises dramatically over the next 30 years, so too will the prevalence of sarcopenia, placing a considerable strain on health care resources (6, 7). Recent studies demonstrate that a diminished muscle myofibrillar protein synthesis (MyoPS) response to protein/amino acid provision in the old may underlie the progression of sarcopenia (8, 9). This age-related muscle anabolic resistance is apparent in response to low- to moderate-dose protein intake (10, 11), common in the diet of older individuals (12). The underlying mechanisms of age-related muscle anabolic resistance are unclear, but are essential to understand to develop interventions to maintain/improve musculoskeletal health.

Although the etiology of sarcopenia is undoubtedly multifactorial, there is contention as to the role of chronological and biological aging in this process (13). It is well accepted that chronological aging is accompanied by declining physical activity levels, interspersed by protracted disuse events (*i.e.*, during illness/hospitalization) (14, 15). This is noteworthy, as it has been demonstrated that bed rest (16), limb immobilization (17), and reduced daily activity (18) induce muscle anabolic resistance and atrophy in older individuals, whereas acute treadmill walking restores postprandial muscle anabolic responsiveness (19). Thus, although physical activity represents an important locus of control in age-related MyoPS, very few studies have assessed physical activity levels in conjunction with MyoPS in older individuals and, as such, any positive association between the two is lacking.

Sarcopenia is often accompanied by a concomitant increase in adiposity and intramuscular fat infiltration (myosteatosis) (20, 21), the confluence of which has been associated with a more rapid progression toward frailty and metabolic disease (22, 23). Indeed, a blunted MyoPS response to amino acid administration was recently demonstrated in obese vs normal-weight older males (24). Furthermore, high-fat diets, lipid administration, obesity, and ectopic fat deposition induce whole-body and muscle anabolic resistance in rodents and humans (25–29), although postprandial MyoPS rates are not impaired in nonobese patients with type 2 diabetes (30, 31). Collectively, these data suggest that obesity, but not insulin resistance *per se*, may impair MyoPS, potentially through compromised muscle quality or elevated levels of adipose-derived inflammatory cytokines (32). Currently, the role of myosteatosis, insulin resistance, and inflammation on age-related muscle anabolic resistance is unclear and warrants further investigation.

Therefore, the aim of the current study was to comprehensively assess the physical activity, whole-body/muscle metabolic health, and inflammatory characteristics of (1) young lean (YL), (2) older lean (OL), and (3) older obese (OO) individuals to establish whether, and to what extent, these parameters were associated with MyoPS in the postabsorptive state and in response to moderate-dose protein provision. We hypothesized that postprandial MyoPS rates would be impaired in OL, but more so in OO individuals compared with YL individuals and would therefore be closely associated with levels of physical activity, adiposity, and inflammatory markers.

## Materials and Methods

### Participants

Ten OL and 10 OO individuals were recruited for comparison against 18 YL individuals (Table 1). Participants completed a general health questionnaire and were excluded from participating if they had type 2 diabetes, uncontrolled hypertension, neuromuscular/cardiovascular/metabolic disease, or if they were smokers, losing weight, or taking nonsteroidal anti-inflammatory drugs. Participants were informed of the purpose and methodology of the study prior to providing written consent. Ethical approval was obtained through the NHS Black Country Research Ethics Committee (13/WM/0429). The study conformed to standards set by the Declaration of Helsinki (seventh edition). Three OO participants were omitted from the study due to the detection of one or more exclusion criteria during preliminary assessments (n = 2) and noncollection of muscle biopsy tissue (n = 1).

**Table 1.** **Anthropometric, Body Composition, and Metabolic Health Data**

	YL	OL	OO
Participants (male/female)	10/8	4/6	4/3
Age (y)	25.5 ± 3.7	71.7 ± 6.2*^a^*	69.1 ± 2.6*^a^*
Body mass (kg)	70.1 ± 12.9	64.5 ± 10.9	91.6 ± 15*^a^*^,^*^b^*
BMI (kg ⋅ m^−2^)	23.3 ± 2.4	22.7 ± 2.5	32.9 ± 4*^a^*^,^*^b^*
Fat mass (% of body mass)	24.3 ± 6.5	28.3 ± 7.1	41 ± 2*^a^*^,^*^b^*
Fat mass (kg)	16.8 ± 5.6	17.7 ± 4.4	36.7 ± 6.4*^a^*^,^*^b^*
Leg fat mass (kg)	6.6 ± 2.5	5.8 ± 1.3	9.9 ± 2.1*^a^*^,^*^b^*
Visceral adipose tissue (kg)	0.23 ± 0.15	0.58 ± 0.48	2.76 ± 1.44*^a^*^,^*^b^*
Lean mass (kg)	49.7 ± 10	43 ± 9.6	50.3 ± 8.8
Lean mass (% of body mass)	71 ± 6.3	66.5 ± 7.3	54.8 ± 2.1*^a^*^,^*^b^*
Leg lean mass (kg)	16.7 ± 3.4	14 ± 3.6	17.1 ± 3.4
Leg lean mass (% of body mass)	23.8 ± 2.14	21.6 ± 2.6*^c^*	18.6 ± 1.3*^a^*^,^*^d^*
HOMA-IR	2.28 ± 0.91	2.16 ± 0.98	6.10 ± 3.02*^a^*^,^*^d^*
Fasting plasma glucose (mmol/L)	5.3 ± 0.5	5.9 ± 0.5*^a^*	6.5 ± 0.3*^a^*
Fasting serum insulin (μIU/mL)	9.7 ± 3.7	8.2 ± 3.5	21.1 ± 10.6*^a^*^,^*^b^*
Plasma IL-6 (pg/mL)	0.43 ± 0.29	1.1 ± 0.51*^a^*	1.49 ± 0.71*^a^*
Plasma CRP (mg/L)	0.37 ± 0.14	0.92 ± 0.34	2.34 ± 0.98*^a^*^,^*^b^*
Plasma HbA1C (mmol/mol)	—	37.3 ± 3.9	38.3 ± 3.4
RMR (kcal)	1681 ± 296	1276 ± 353*^c^*	1515 ± 449

Data presented as mean ± standard deviation.

Abbreviation: HOMA-IR, homeostasis model assessment of insulin resistance.

^a^ *P* < 0.01, significantly different from YL.

^b^ *P* < 0.01, significantly different from OL.

^c^ *P* < 0.05, significantly different from YL.

^d^ *P* < 0.05, significantly different from OL.

### Experimental design

Following an initial screening visit, participants reported to the National Institute for Health Research/Wellcome Trust Clinical Research Facility (CRF) of the Queen Elizabeth Hospital, Birmingham, United Kingdom, over two visits for preliminary assessments of dietary intake, physical activity, body composition, and whole-body indices of metabolic health/inflammation. During a third visit, participants underwent a stable amino acid isotope tracer infusion with serial muscle biopsies and blood samples to determine MyoPS rates in the basal postabsorptive state and in response to a moderate-dose milk protein isolate drink (hereafter termed postprandial), intracellular signaling, and muscle fiber/intramyocellular lipid (IMCL) properties.

### Preliminary assessments

#### Body composition

Participants reported to the CRF at ∼0800 h, following a 10-hour overnight fast. Following assessment of height and weight, dual energy x-ray absorptiometry scanning (GE iDXA; GE Healthcare, Chicago, IL) was used to determine whole-body and regional fat mass, lean mass, and visceral adipose tissue (VAT). VAT was obtained by identifying the android/gynoid region and the horizontal plane by the area between the pelvis and the rib cage, with its vertical limits being the inner abdominal muscle wall. VAT is computed by subtracting subcutaneous fat from the total android fat mass in the android region. Obesity was defined by body mass index (BMI) ≥30 kg ⋅ m*^−^*^2^.

#### Resting metabolic rate

Participants rested in a supine position for 30 minutes while expired gases were collected via a ventilated plastic hood and analyzed for O_2_ and CO_2_ concentrations using a Moxus metabolic cart (AEI Technologies, Naperville, IL), equipped with electrochemical gas analyzers (AME-TEK model S-3A/1 and CD-3A). Breath-by-breath measurements were analyzed for volume of oxygen (VO_2_) and volume of CO_2_ (VCO_2_) and values averaged over 30-second periods using Moxus software (version 2.8.02). Participants were instructed to lie as still as possible, breathe normally, not speak, and stay awake. Resting metabolic rate (RMR) was predicted using the Weir equation [RMR = (3.94 × VO_2_) + (1.11 × VCO_2_)] based upon a 5-minute measurement period during which VO_2_ measurements showed the lowest coefficient of variation.

#### Fasting plasma blood glucose and hemoglobin A1C

Following RMR assessment, a 10-mL venous blood sample was obtained to measure fasting plasma glucose and hemoglobin A1C concentrations. Participants were excluded from study participation if fasting plasma glucose and hemoglobin A1C values exceeded 7 mmol/L and 48 mmol/mol, respectively, indicating high risk or presence of type 2 diabetes.

#### Physical activity/intensity levels

Participants were fitted with a wrist-worn accelerometer with a triaxial sensor (GENEActiv; Activinsights, Huntingdon, United Kingdom) for 4 consecutive days, including at least 1 weekend day. Accelerometers were initialized to sample data at a 10-Hz frequency. Data were converted into 60-second epochs and analyzed using the GENEActiv software (version 2.2; Activinsights). Activities were split into four categories based on metabolic equivalent (MET) values: (1) sedentary activity (<1.5 METs), (2) light activity (1.5 to 3.99 METs), (3) moderate activity (4.0 to 6.99 METs), and (4) vigorous activity (>7 METs) (33). Participants also recorded their daily step count over 4 consecutive days using a hip-worn pedometer (Vital Steps; Omron Healthcare Co. Ltd., Milton Keynes, United Kingdom).

#### Dietary intake

Habitual dietary intake was assessed over a 4-day period. Participants were given a food diary and digital balance scales to record and weigh out their dietary and fluid intake. Food diaries were analyzed using Dietplan software (Dietplan 7; Forestfield Software Ltd, West Sussex, United Kingdom).

### Experimental trial

The evening prior to the experimental trial, participants consumed a standardized evening meal (787 kcal) composed of ∼19% protein, ∼46% carbohydrate, and ∼35% fat. After an ∼10- to 12-hour overnight fast and having refrained from any strenuous activity for 48 hours previously, participants reported to the CRF at 0730 hours by car or public transport. Upon arrival, a 21-gauge cannula was inserted into an antecubital vein of both forearms for serial blood sampling throughout the day and administration of a primed continuous infusion of l-[ring-^13^C_6_] phenylalanine (prime dose 2 μmol ⋅ kg^−1^; continuous infusion 0.05 μmol ⋅ kg^−1−^⋅ min^−1^; Cambridge Isotope Laboratories, Andover, MA), initiated after obtainment of a baseline blood sample. Arterialized blood samples were drawn at −180, −95, −35, and −5 minutes prior to and 20, 40, 60, 90, 120, 180, and 240 minutes after a milk protein beverage from a heated forearm vein (60°C). Blood samples were collected in serum separator and EDTA-treated vacutainers (BD Biosciences, Oxford, United Kingdom) and centrifuged at 3000 rpm for 10 minutes at 4°C, with plasma and serum aliquots stored at −80°C. After 150 minutes of infusion, a muscle biopsy was obtained from quadriceps *vastus lateralis* of a randomly selected leg under local anesthesia (1% lidocaine) using the Bergström technique. Biopsy samples were freed from visible blood, adipose, and connective tissue using ice-cold saline, snap-frozen in liquid nitrogen, and stored at −80°C for analysis of MyoPS and intramuscular signaling. A separate piece of muscle tissue (∼20 mg) was mounted in O.C.T. compound (VWR Chemicals, Leuven, Belgium) and frozen in 2-methylbutane (Sigma-Aldrich, St. Louis, MO) before being stored at −80°C for immunohistochemical analyses. Immediately after biopsy obtainment, participants consumed a 15-g dose of milk protein isolate (MyProtein, Cheshire, United Kingdom) dissolved in 300 mL water. The amino acid content of the milk protein isolate was (as percentage content): alanine, 3.0; arginine, 3.7; asparagine, 7.4; cysteine, 0.9; glutamine, 9.1; glycine, 1.7; histidine, 2.3; isoleucine, 5.6; leucine, 9.8; lysine, 8.7; methionine, 2.6; phenylalanine, 4.7; proline, 9.9; serine, 5.3; threonine, 3.8; tryptophan, 1.7; tyrosine, 4.6; and valine, 6.0, providing 13.6 g amino acids in total. A small amount of l-[ring-^13^C_6_] phenylalanine tracer was added to the drink to minimize changes in ^13^C_6_ phenylalanine enrichment (28.2 mg to enrich to 4%) (34). Approximately 240 minutes after drink consumption, a second biopsy was obtained ∼3 cm proximal to the first biopsy and indicated the end of the experimental trial. Participants were fed a meal of their choice and monitored for 30 minutes before leaving the CRF.

### Blood analyses

#### Blood analyte and hormone concentrations

Plasma glucose was analyzed by enzymatic colorimetric assay (Glucose Oxidase kit; Instrumentation Laboratory, Warrington, United Kingdom) using an ILAB 650 Clinical Chemistry Analyzer (Instrumentation Laboratory). Plasma HbA1C concentration was analyzed via a Tosoh G8 HPLC semiautomated analyzer (Tosoh Bioscience, King of Prussia, PA). Serum interleukin-6 (IL-6), C-reactive protein (CRP), and insulin concentrations were measured using commercially available enzyme-linked immunosorbent assay kits (IL-6: R&D Systems Europe, Abingdon, United Kingdom; CRP: RayBiotech, Norcross, GA; insulin: IBL International, Hamburg, Germany).

#### Plasma amino acid enrichment and concentrations

Plasma ^13^C_6_ phenylalanine enrichment was determined by gas chromatography mass spectrometry alongside leucine and phenylalanine concentrations, as previously described (35).

### Muscle tissue analyses

#### Myofibrillar protein enrichment and intramuscular signaling

Approximately 30 to 40 mg of muscle tissue was minced in 10 μL/mg of homogenization buffer (50 mM Tris-HCL, 1 mM EDTA, 1 mM EGTA, 10 mM *β*-glycerophosphate, 50 mM sodium fluoride, and one protease inhibitor tablet per 10 mL of homogenization buffer, pH 7.5) on ice using small scissors and a Teflon pestle before being shaken for 10 minutes at 1500 rpm at room temperature and centrifuged at 11,000 rpm at 4°C for 5 minutes. The supernatant containing the sarcoplasmic proteins was transferred to a 2-mL eppendorf for western blotting according to our previous work (35). The remaining myofibrillar pellet was processed and analyzed for ^13^C_6_ enrichment according to our previous work (35). Primary antibodies used for western blotting were: phospho p70S6K1 Thr389 (#9205), total p70S6K1 (#9202), phospho–eukaryotic initiation factor 4E binding protein (4E-BP1) Thr37/46 (#9459), total 4E-BP1 (#9452), phospho–eukaryotic elongation factor 2 (p-eEF2) Thr56 (#2331), total eEF2 (#2332), phospho–protein kinase B (Akt) Ser473 (#3787), and total Akt (#9272) from Cell Signaling Technology (New England Biolabs Ltd, Hitchin, United Kingdom).

#### Fiber-type IMCL

Muscle cross sections (5 µm) were fixed for 1 hour in 3.7% formaldehyde, washed briefly in double-distilled H_2_O, permeabilized for 5 minutes in 0.5% Triton X-100, and washed three times for 5 minutes in 1× phosphate-buffered saline (PBS). Cross sections were then incubated for 2 hours in myosin heavy chain type I antibody [A4.840, DHSB; developed by Dr. H. M. Blau (Baxter Lab for Stem Cell Biology, Stanford University, CA), IA (1:25 diluted in 5% normal goat serum and PBS)], washed three times for 5 minutes in PBS, and subsequently incubated for 30 minutes in goat anti-mouse IgM 594 [#A21044; Thermo Fisher Scientific, Leicestershire, United Kingdom (1:50 diluted in PBS)]. After three 5-minute washes in PBS, sections were incubated for 30 minutes in wheat germ agglutinin stock solution [W11263; Alexa Fluor 350; Thermo Fisher Scientific (1 mg/mL, diluted 1:100 in PBS)] and washed two times for 3 minutes in PBS. Finally, sections were incubated for 20 minutes in bodipy-493/503 stock solution [D3922; Thermo Fisher Scientific (1 mg/mL, diluted 1:50 in PBS)] and washed three times for 3 minutes in PBS. Coverslips were applied using a mounting solution containing glycerol and mowiol 4-88 solution (Sigma-Aldrich) in 0.2 M Tris buffer (pH 8.5) with 0.1% 1,4-diazabicyclo-[2,2,2]-octane (DABCO) antifade medium. Images were taken with an Eclipse E600 (Nikon, Badhoevedorp, the Netherlands) and a 40× zoom and analyzed using Pro Suite version 5.1.2.59.

### Calculations

The precursor-product method was used to calculate MyoPS rates from ^13^C_6_ phenylalanine incorporation:

$$FSR\left(\%\cdot h^{\mathrm{-1}}\right)=\Delta E_{b}/E_{p}\times 1/t\times 100$$

where *Δ E_b_* is the algebraic difference in bound ^13^C_6_ phenylalanine between biopsy samples, *E_p_* is the precursor enrichment (mean plasma ^13^C_6_ phenylalanine enrichment from arterialized blood), and *t* is the time between biopsy samples (in minutes). Preinfusion plasma ^13^C_6_ phenylalanine enrichment was used as a proxy for basal muscle protein enrichment, as previously validated in young and older individuals (34, 36).

### Statistics

Data analysis was performed using SPSS version 22 (IBM, Chicago, IL) and Prism 6 (GraphPad Software, La Jolla, CA). A Shapiro-Wilks test was used to test the normality of the data. Subsequently, nonnormally distributed variables were logarithmically transformed. MyoPS rates and intracellular signaling were analyzed using a two-way, repeated-measures analysis of variance (ANOVA) with one within (two levels—postabsorptive and postprandial) and one between factor (three levels; group). Insulin, plasma amino acid, and ^13^C_6_ phenylalanine enrichment were analyzed using a one-way, repeated-measures ANOVA (time × group). Linear regression was performed on ^13^C_6_ phenylalanine enrichments to assess the existence of any deviation in enrichment. All other data points were analyzed using a one-way ANOVA. Tukey honest significant difference *post hoc* analysis was performed whenever a noteworthy F ratio was found to isolate specific differences. Correlational analysis was performed using a two-tailed Pearson correlation coefficient test or Spearman test (for nonnormally distributed data). Significance for all analyses was set at *P* < 0.05. All values are presented as means ± standard deviation or standard error of the mean.

## Results

### Anthropometric and metabolic health characteristics

Body mass, BMI, fat mass, and VAT were significantly greater in OO compared with OL and YL (*P* < 0.05), with no differences between YL and OL. The whole-body absolute lean mass and lower limb absolute lean mass were not significantly different between groups. Whole-body lean mass, expressed relative to total body mass, was significantly lower in OO compared with YL and OL (*P* < 0.001 and *P* = 0.001, respectively). Relative leg lean mass was lower in OO and OL compared with YL (*P* < 0.001 and *P* = 0.038, respectively) and lower in OO compared with OL (*P* = 0.023). Plasma IL-6, CRP, glucose, serum insulin, and homeostasis model assessment of insulin resistance were significantly greater in OO compared with OL and YL (*P* < 0.01). RMR was significantly lower in OL compared with YL only (*P* = 0.015). Anthropometric and metabolic health characteristics are presented in Table 1.

### Physical activity and dietary characteristics

Average daily step count was significantly lower in OO compared with OL and YL (*P* < 0.01). Relative and absolute time spent in light intensity activity was significantly greater in OL compared with YL (*P* = 0.003), but not different from OO. Dietary protein intake was significantly lower in OO compared with YL (*P* = 0.009), but not different from OL. Physical activity and dietary characteristics are detailed in Table 2.

**Table 2.** **Activity and Diet Characteristics**

	YL	OL	OO
Daily step count	11,870 ± 3905	11,102 ± 3271	5399 ± 2079*^a^*^,^*^b^*
Sedentary activity (%)	70.8 ± 6.6	65.5 ± 13.8	74.9 ± 7.4
Light activity (%)	10.2 ± 2.1	15 ± 5.2*^c^*	11 ± 2.4
Moderate activity (%)	16.8 ± 5.2	18.4 ± 9.7	13.9 ± 5.9
Vigorous activity (%)	2.3 ± 2.8	0.8 ± 1.5	0.2 ± 0.1
Sedentary activity (min)	643 ± 56	594 ± 115	684 ± 104
Light activity (min)	93 ± 19	140 ± 50*^c^*	101 ± 24
Moderate activity (min)	153 ± 53	169 ± 89	126 ± 48
Vigorous activity (min)	21 ± 26	7 ± 14	2 ± 1
Daily energy intake (kcal)	2162 ± 473	2091 ± 451	2412 ± 628
Daily protein (g ⋅ kg^−1^)	1.51 ± 0.46	1.36 ± 0.37	0.95 ± 0.20*^a^*
Daily CHO (g ⋅ kg^−1^)	3.20 ± 1.22	3.38 ± 0.91	2.65 ± 1.25
Daily fat (g ⋅ kg^−1^)	1.17 ± 0.33	1.29 ± 0.46	1.07 ± 0.40
Daily alcohol (g ⋅ kg^−1^)	0.22 ± 0.22	0.22 ± 0.25	0.33 ± 0.25
Daily fiber (g ⋅ kg^−1^)	0.30 ± 0.14	0.34 ± 0.10	0.24 ± 0.12

Values presented as mean ± standard deviation.

^a^ *P* < 0.01, significantly different from YL.

^b^ *P* < 0.01, significantly different from OL.

^c^ *P* < 0.05, significantly different from YL.

### Muscle fiber characteristics and lipid content

Type I and II muscle fiber cross-sectional area (CSA) was greater in YL compared with OO and OL (*P* < 0.001 for both) and significantly greater in OO compared with OL (*P* = 0.004 and *P* < 0.001, respectively). Type I fiber lipid droplet number was significantly greater in YL compared with OL (*P* < 0.001) and OO (*P* = 0.021) and was greater in OL compared with OO (*P* < 0.001). Type II fiber lipid droplet number was significantly greater in YL and OO compared with OL (*P* < 0.001 for both) with no difference between YL and OO. Type I lipid droplet area (normalized to fiber CSA) was lower in OL compared with YL (*P* = 0.014) but not OO, whereas type II lipid droplet area was significantly greater in OO compared with YL (*P* < 0.001) and OL (*P* = 0.042). Muscle fiber and IMCL characteristics are presented in Table 3.

**Table 3.** **Muscle Fiber and IMCL Characteristics**

	YL	OL	OO
Type I fiber (%)	48 ± 16	49 ± 18	36 ± 15
Type II fiber (%)	52 ± 16	51 ± 18	65 ± 16
Type I area (μm^2^)	4031 ± 1978	3009 ± 1251*^a^*	3421 ± 1528*^a^*^,^*^b^*
Type I lipid droplet number	616 ± 458	412 ± 298*^a^*	533 ± 505*^b^*^,^*^c^*
Type I lipid droplet area (μm^2^)	287 ± 238	175 ± 159*^a^*	244 ± 267*^b^*^,^*^c^*
Type I lipid droplet area (% fiber area)	0.077 ± 0.06	0.064 ± 0.063*^a^*	0.069 ± 0.065
Type II area (μm^2^)	4009 ± 1733	2170 ± 1243*^a^*	3390 ± 1199*^a^*^,^*^b^*
Type II lipid droplet number	377 ± 399	242 ± 312*^a^*	415 ± 407*^b^*
Type II lipid droplet area (μm^2^)	133 ± 157	76 ± 99*^a^*	201 ± 207*^a^*^,^*^b^*
Type II lipid droplet area (% fiber area)	0.031 ± 0.028	0.032 ± 0.034	0.058 ± 0.054*^a^*^,^*^b^*

Data presented as mean ± standard deviation.

^a^ *P* < 0.01, significantly different from YL.

^b^ *P* < 0.01, significantly different from OL.

^c^ *P* < 0.05, significantly different from YL.

### Plasma amino acid and insulin concentrations

**Figure 1.**
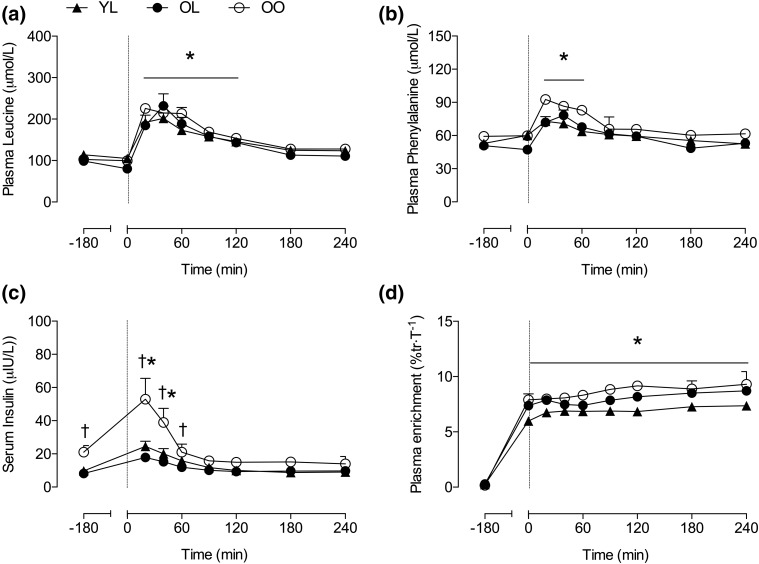
(a) Plasma leucine concentration, (b) phenylalanine concentration, (c) serum insulin concentration, and (d) plasma ^13^C_6_ phenylalanine enrichment in experimental trials. At *t* = 0, a 15-g bolus of milk protein isolate was consumed. Values are means ± standard error of the mean. Significance was set at *P* < 0.05. *Significantly greater than fasting values (−180 min); ^†^significantly greater in OO compared with other groups.

Plasma leucine and phenylalanine concentrations increased above basal-fasted values after ingestion of 15 g of milk protein isolate [*P* < 0.001; Fig. 1(a) and 1(b)], peaking between 20 and 40 minutes post-ingestion and returning to basal-fasted values by 180 and 90 minutes postingestion, respectively, with no difference between groups. Basal-fasted serum insulin concentration was significantly elevated in OO compared with YL and OL (*P* < 0.001). Serum insulin levels increased above basal-fasted values at 20 minutes post-ingestion (*P* < 0.01 for all), returning to basal-fasted values by 60 minutes post-ingestion [Fig. 1(c)]. Absolute serum insulin values at 20 and 40 minutes after protein ingestion were greater in OO compared with YL (*P* = 0.004 and 0.016, respectively) and OL (*P* = 0.001 and 0.007, respectively), with no difference between YL and OL. Plasma ^13^C_6_ phenylalanine enrichment increased above basal (−180 minutes) values 60 minutes after the initiation of the stable isotope tracer infusion (*P* < 0.001) and remained stable for the entire trial duration [Fig. 1(d)]. The slopes of the plasma ^13^C_6_ phenylalanine enrichment were not significantly different from zero, indicating the presence of an isotopic steady state (*P* = 0.3).

### MyoPS and intramuscular signaling

**Figure 2.**
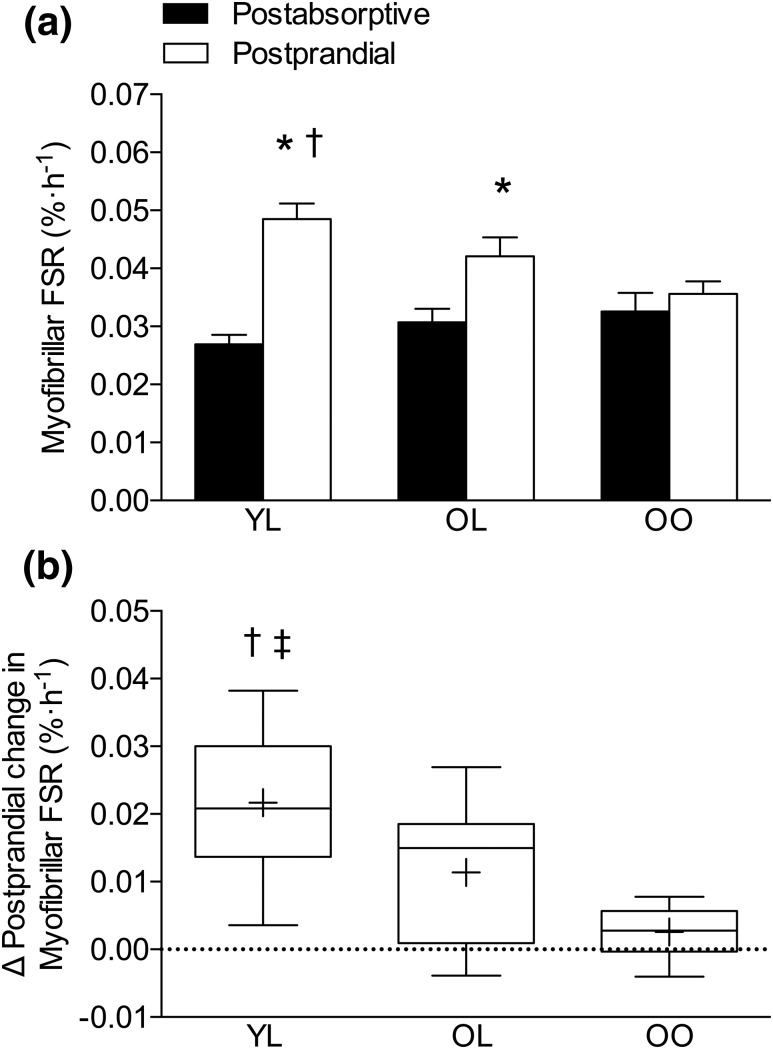
(a) Myofibrillar fractional synthesis rate (FSR) in rested postabsorptive and 4-h postprandial state following ingestion of 15 g of milk protein (dashed line at 0 min), with values presented as means ± standard error of the mean. (b) Net postprandial change in myofibrillar FSR from postabsorptive values, showing the median (central horizontal line), 25th and 75th percentiles (box), minimum and maximum values (vertical lines), and mean (cross). Significance was set at *P* < 0.05. *Significantly greater than corresponding postabsorptive values; ^†^significantly greater than OO; ^‡^significantly greater than OL.

Postabsorptive MyoPS rates did not differ between groups. Following ingestion of 15 g of milk protein isolate, MyoPS rates increased by ∼81% in YL (*P* < 0.001) and ∼38% in OL (*P* = 0.002), respectively, with no notable increase found in OO (*P* = 0.11). Postprandial MyoPS rates were significantly greater in YL compared with OO (*P* = 0.02), but were not different between YL and OL (*P* = 0.21) or between OL and OO [*P* = 0.11; Fig. 2(a)]. The net postprandial MyoPS response, expressed as the delta change from postabsorptive rates, was significantly greater in YL compared with OL (*P* = 0.032) and OO (*P* < 0.001), with no considerable difference found between OL and OO [*P* = 0.173; Fig. 2(b)]. Phosphorylation of p-70S6K^Thr389^, Akt^Ser473^, eEF2^Thr56^, and 4E-BP1^Thr37/46^ was not significantly different from basal-fasted values at 240 minutes after protein consumption and was not different between groups at any time point (Supplemental Figs. 1 and 2).

### Correlations

**Figure 3.**
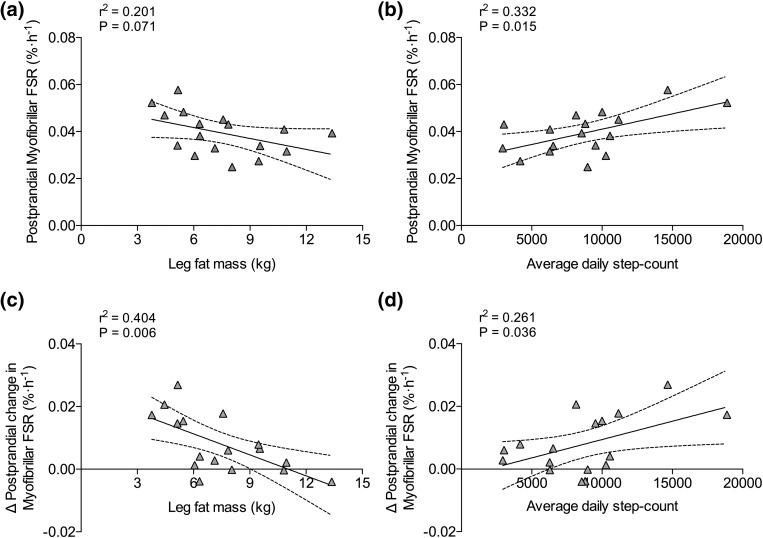
Correlations between (a) absolute postprandial myofibrillar fractional synthesis rate (FSR) and leg fat mass and (b) average daily step count in OL and OO combined. Correlations between (c) the delta change in postprandial myofibrillar FSR from postabsorptive values and leg fat mass and (d) average daily step count in OL and OO combined.

Absolute postprandial MyoPS rates showed a tendency to correlate negatively with leg fat mass [*r*^2^ = 0.20; *P* = 0.07; Fig. 3(a)] and correlated positively with average daily step count in OL and OO combined [*r*^2^ = 0.33; *P* = 0.015; Fig. 3(b)]. The net postprandial MyoPS response (*i.e.*, delta change from postabsorptive values) correlated negatively with leg fat mass [*r*^2^ = 0.4; *P* = 0.006; Fig. 3(c)] and positively with average daily step count for OL and OO combined [*r*^2^ = 0.26; *P* = 0.036; Fig. 3(d)]. HOMA-IR correlated positively with type I fiber lipid droplet number (*r*^2^ = 0.41; *P* = 0.018), type I fiber lipid droplet area (*r*^2^ = 0.47; *P* = 0.009), leg fat mass (*r*^2^ = 0.4; *P* = 0.007), and average daily step count (*r*^2^ = 0.26; *P* = 0.036) for OL and OO combined. Although not significant, there was a trend for a negative correlation between the net postprandial MyoPS response and serum CRP in older individuals (*r*^2^ = 0.23; *P* = 0.050).

## Discussion

Age-related impairment in the muscle protein synthetic response to protein-based nutrition is thought to be the primary metabolic defect underpinning the progression of sarcopenia (10). To what extent this age-related muscle anabolic resistance is a consequence of chronological and/or aspects of biological aging is unknown. The present data demonstrate that MyoPS rates are increased above postabsorptive values following moderate-dose milk protein ingestion in YL and OL, but not OO individuals. Although there was no difference in absolute postprandial MyoPS rates between YL and OL, the net postprandial increase in MyoPS rates (delta change from postabsorptive rates) was greater in YL compared with both OL and OO. Postprandial MyoPS rates (absolute and delta change from postabsorptive) were associated with leg fat mass and average daily step count in older individuals. Collectively, these data demonstrate that muscle anabolic resistance to moderate-dose protein ingestion is apparent in healthy old age, but is exacerbated in obese individuals with low physical activity.

In the current study, we provided participants with an oral 15-g bolus of milk protein isolate (∼0.3, 0.35, and 0.3 g ⋅ kg^−1^ ⋅ lean mass^−1^ for YL, OL and OO, respectively), based on evidence that age-related muscle anabolic resistance may be most apparent with low-to-moderate protein doses typically consumed in the diet (10, 12). This feeding strategy produced postprandial MyoPS responses that were ∼81% and 38% greater than postabsorptive values in YL and OL, respectively. Although not statistically different, absolute postprandial MyoPS rates were 13% lower in OL vs YL. Furthermore, we observed that the increase in MyoPS above postprandial values was significantly lower in OL vs YL. We acknowledge that the differences in net postprandial MyoPS response between OL and YL we observe in this study may differ with high-dose protein intake (≥30 g) or in the context of a mixed-macronutrient meal. In line with our findings, others have reported that ingestion of 20 g of casein protein increases muscle protein synthesis by ∼75% and 21% in healthy lean young and older males, respectively, with absolute postprandial protein synthesis rates being ∼16% lower in old vs young (37). Additionally, our observations reveal the inability of OO to increase postprandial MyoPS above postabsorptive values. Absolute postprandial and net change in postprandial MyoPS rates were not statistically lower in OO vs OL, likely due to the relatively small sample size. However, Murton *et al*. (24) recently demonstrated an impaired postprandial MyoPS in obese vs normal-weight older males. Collectively, our findings provide evidence of age-related muscle anabolic resistance to moderate-dose protein ingestion. Furthermore, the associations between absolute and net postprandial MyoPS responses and leg fat mass/step count suggest that age-related muscle anabolic resistance is exacerbated in obese inactive older individuals.

Age-related muscle loss is commonly accompanied by a concomitant increase in whole-body adiposity and ectopic fat deposition within skeletal muscle (20, 21), which has been associated with accelerated sarcopenia progression and metabolic disease risk (23). It has been postulated that obesity-induced blunting of postprandial MyoPS might be due to IMCL accumulation (24) or adipose tissue–derived inflammatory cytokines (adipokines) (38). Alongside the negative association between the net postprandial MyoPS response and leg fat mass, we observed a trend for a similar negative association with fasting serum CRP, but were unable to demonstrate any association with fiber-specific IMCL content, despite twofold higher type II fiber IMCL in OO compared with OL and YL. This was somewhat surprising given previous reports of associations between IMCL and muscle anabolic resistance in old rodents (28). In our hands, type I fiber IMCL content was equivalent between YL and OO. However, type I fiber IMCL content was negatively associated with insulin sensitivity for OL and OO, but not YL, reinforcing the notion that the capacity for IMCL oxidation would likely have been greater in YL (39). Thus, although we did not observe any association of fiber-type IMCL content with the postprandial change in MyoPS, the subcellular location of IMCL and/or specific class of lipid intermediates (*i.e.*, ceramides and diacylglycerol) may associate more closely with age-related muscle anabolic resistance.

Despite the near-completely diminished postprandial MyoPS response in OO, there was no considerable difference in whole-body or regional lean mass compared with OL and YL. In fact, muscle fiber CSA was preserved to a greater extent in OO than OL, although still reduced compared with YL. Furthermore, the lower RMR in OL vs YL and OO may be explained by marginally lower whole-body lean mass. In line with our findings, others have reported greater lean mass in obese vs normal-weight older and younger individuals (24, 40). These data refute the mechanistic role of muscle anabolic resistance in sarcopenia and support the existence of an age-associated obesity paradox, by which obesity might protect against sarcopenia (41). Collectively, these data beg the question, when, if at all, does obesity-induced muscle anabolic resistance manifest in an accelerated loss of skeletal muscle in older individuals? We postulate that the extra work necessary during locomotion and daily living in OO might offer a degree of protection against sarcopenia up to a point, beyond which the decline in muscle mass may be precipitous. Progression toward this brink may, therefore, be influenced by muscle quality (*i.e.*, inter- and perhaps intramuscular adiposity) rather than quantity. Furthermore, the greater insulin resistance in OO compared with OL could imply that the quality of muscle tissue retained in old age, and not necessarily the quantity, may play a more relevant role in whole-body metabolic health.

Physical activity is an important locus of control in the regulation of age-related muscle protein turnover. In this study, we demonstrate that YL and OL took ∼122% and 106% more daily steps compared with OO, respectively. Interestingly, postprandial MyoPS (absolute and net increase above postabsorptive values) correlated significantly with average daily step count in older individuals, which aligns with evidence that reduced physical activity elicits muscle anabolic resistance in older individuals (18). However, the net postprandial MyoPS response was still lower in OL compared with YL, despite equivalent daily physical activity levels, suggesting that increasing average daily step count in OO, to a level observed in OL may, at best, only partially restore postprandial MyoPS responses. Indeed, an acute bout of moderate-intensity treadmill walking has been reported to enhance postprandial muscle protein synthesis rates in older males (19). The addition of activity-matched OO and OL individuals would have allowed us to reconcile this important point, but was beyond the scope of the current study. We observed no difference between groups in the relative time spent at different activity intensities or any association between physical activity intensities and postprandial MyoPS responses. However, combined time spent in light plus moderate plus vigorous intensity activity revealed a deficit of ∼38 and 87 minutes for OO compared with YL and OL, respectively, which might explain the lower average daily step count in OO [assuming an average of ∼100 steps ⋅ min^−1^ and slower preferred walking speed (42, 43)]. Thus, our findings suggest a minimum number daily steps (or time spent active) might offer partial protection against age-related muscle anabolic resistance.

In conclusion, we have demonstrated that chronological aging reduces the net postprandial MyoPS response to moderate-dose protein ingestion, whereas obesity and inactivity exacerbate, and are associated with, this muscle anabolic resistance. Despite the negative association between leg fat mass and the net postprandial MyoPS response in older individuals, we were unable to demonstrate a similar association with fiber-type IMCL content. Interestingly, despite evidence of exacerbated muscle anabolic resistance in OO, absolute lean tissue mass was equivalent and muscle fiber area greater than OL, which questions the mechanistic role of muscle anabolic resistance in sarcopenia. As such, further work is required to delineate the precise mechanisms through which obesity and inactivity exacerbate sarcopenia, particularly in very old individuals. There is also a need to understand whether the subcellular location of IMCL are associated with age-related muscle anabolic resistance. Although we did not directly address sex-based alterations in MyoPS, there was no discernible pattern of difference between older men and women. Nonetheless, others have demonstrated sexual dimorphism in muscle protein synthesis in older individuals (44), and this issue warrants further clarification. Finally, our data suggest that physical activity may partially protect against muscle anabolic resistance in older individuals, although further work is required to determine whether chronic physical activity interventions can restore muscle protein anabolism in normal-weight and obese older individuals.
