# Supplementary figures and images for: Age-Related Anabolic Resistance of Myofibrillar Protein Synthesis Is Exacerbated in Obese Inactive Individuals
Source: J Clin Endocrinol Metab. 2017 Jul 14;102(9):3535–45. doi: 10.1210/jc.2017-00869 (PMC5587073; doi:10.1210/jc.2017-00869)

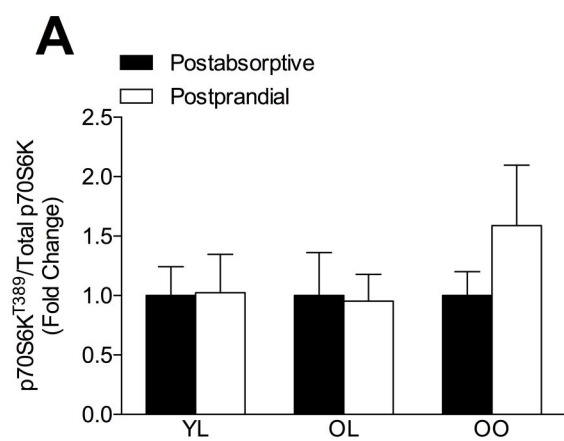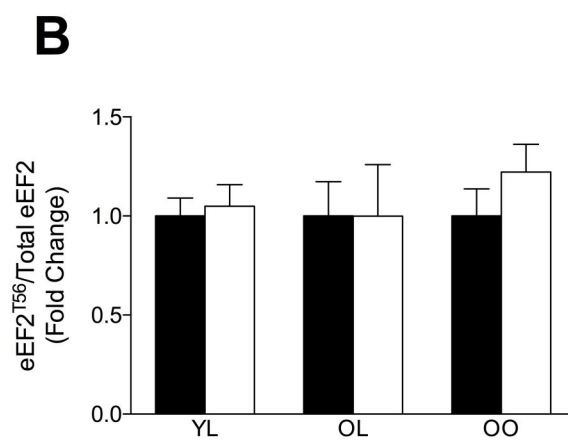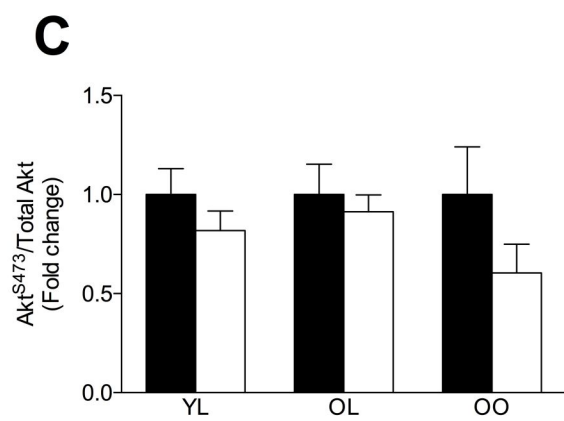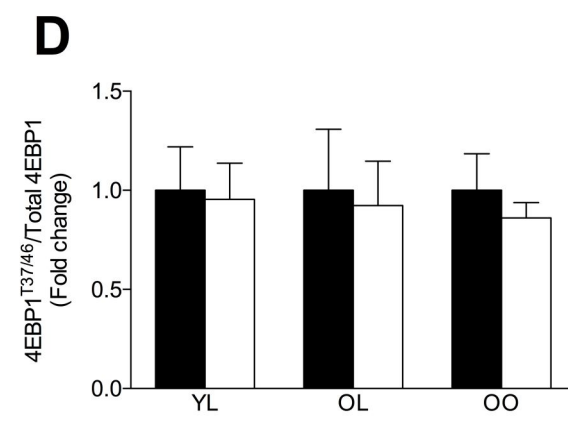

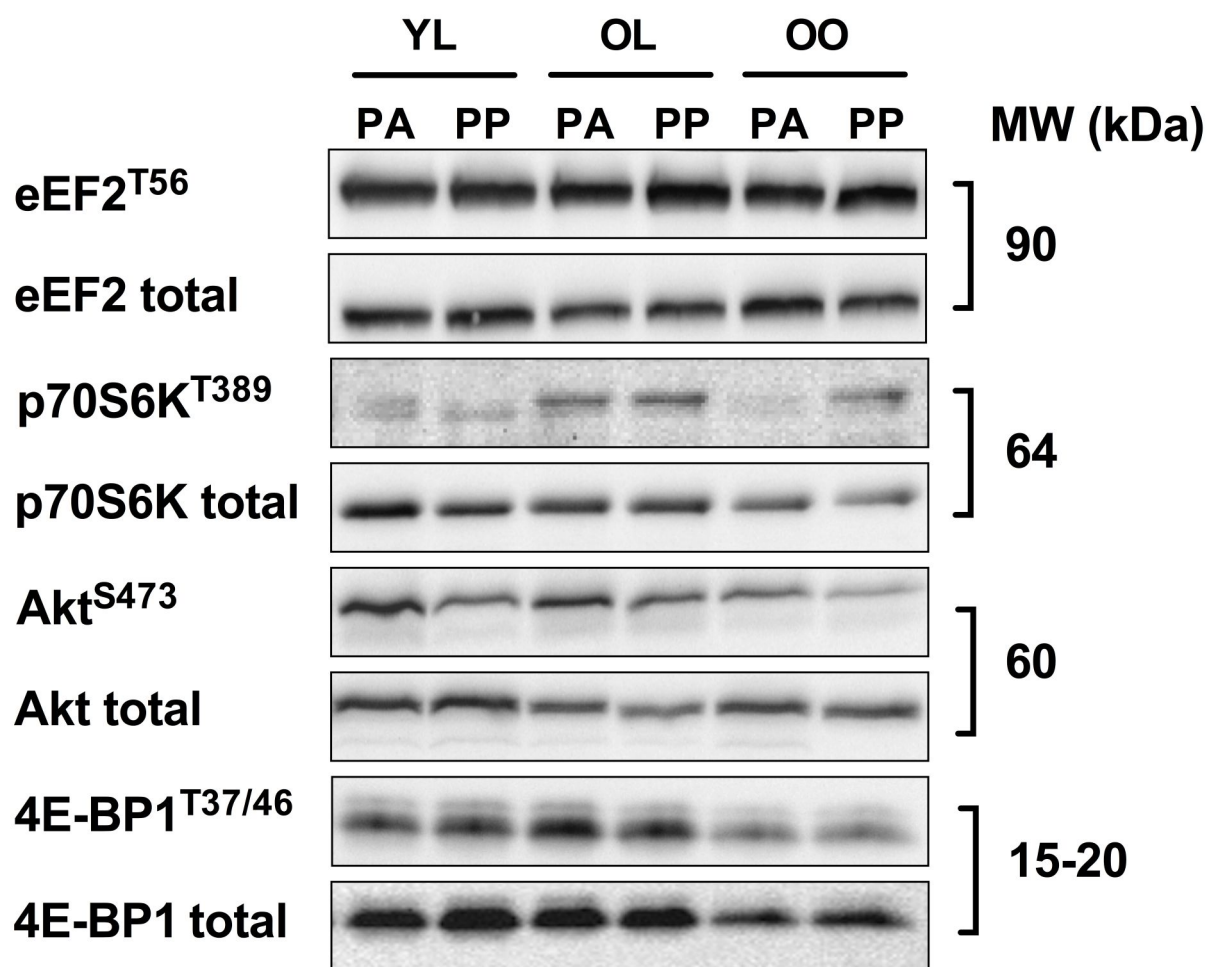

Supplement: Supplementary file 1 [file jc.2017-00869.sf1.pdf]
